# Single-cell analyses to tailor treatments

**DOI:** 10.1126/scitranslmed.aan4730

**Published:** 2017-09-20

**Authors:** Alex K. Shalek, Mikael Benson

**Affiliations:** 1 Institute for Medical Engineering & Science (IMES) and Department of Chemistry, Massachusetts Institute of Technology (MIT), Cambridge, MA 02139, USA; 2 Broad Institute of MIT and Harvard, Cambridge, MA 02142, USA; 3 Ragon Institute of Massachusetts General Hospital, MIT, and Harvard, Cambridge, MA 02139, USA; 4 The Centre for Personalised Medicine and Division of Pediatrics, Department of Clinical and Experimental Medicine, Linköping University, 58183 Linköping, Sweden

## Abstract

Single-cell RNA-seq could play a key role in personalized medicine by facilitating characterization of cells, pathways, and genes associated with human diseases such as cancer.

We discuss the potential of single-cell RNA sequencing (scRNA-seq) to empower clinical implementation of personalized medicine. On the basis of work to date, we emphasize applications in cancer. Nevertheless, the underlying problems and solutions should be generally applicable to other complex diseases (*1*, *2*).

One of health care’s largest outstanding issues is that many patients do not respond to treatment. By recent estimates, about 90% of drugs are effective for less than 50% of patients (*3*). This causes enormous physical, social, and economic suffering. The annual cost of ineffective treatment is estimated at $350 billion/year in the United States alone. Moreover, variable treatment response contributes to the rising cost of drug development, currently around $2.6 billion per drug. A fundamental driver of these inefficiencies is the cellular heterogeneity that exists within and between patients in cancer and other complex diseases, which can involve altered behaviors across multiple cell types and hundreds of genes (*1*, *2*). To date, such changes have often been profiled at the population level. This can mask intercellular variations that can be functionally and clinically relevant (*4*). For instance, in cancer, treatment failure may result if a tumor contains many malignant subsets, of which only some respond to treatment (Fig. 1, A and B) (*1*, *4*).

**Fig. 1:**
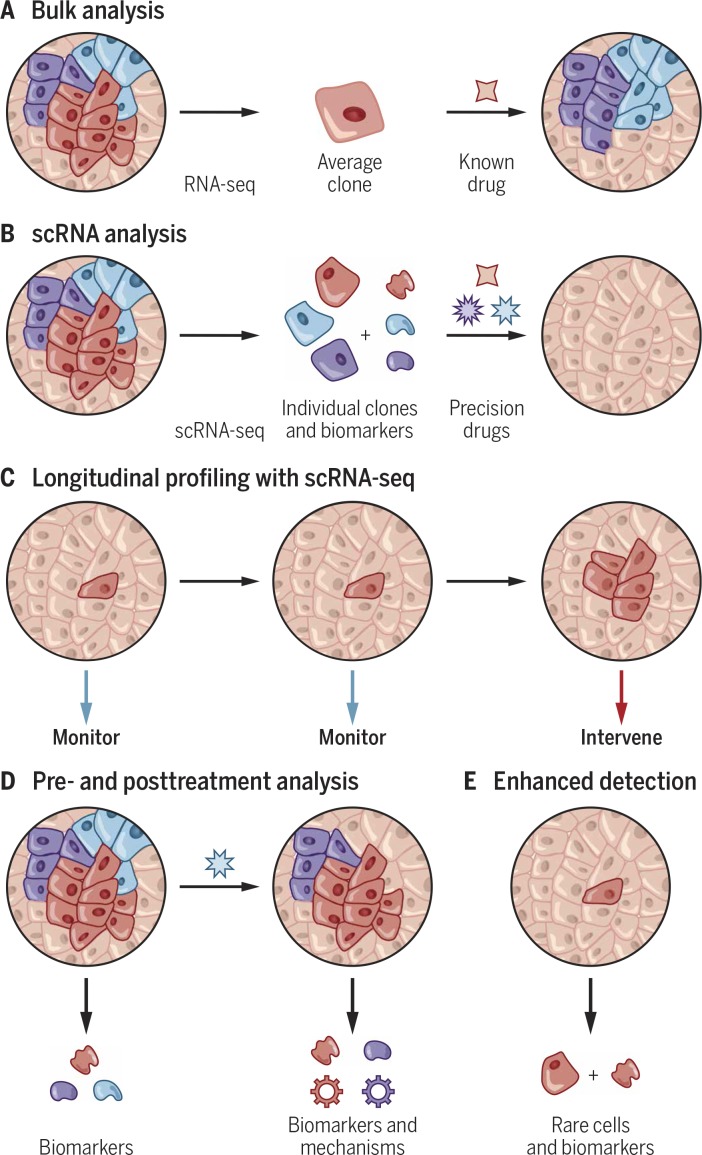
**scRNA-seq applications in cancer medicine.** (**A**) Bulk analysis of a tumor identifies the predominant malignant clone and suggests a drug to target it but not other clones. (**B**) scRNA-seq resolves each clone within a tumor, as well as the corresponding biomarkers and cognate drugs, enabling successful therapy. (**C**) Longitudinal profiling of patient samples with scRNA-seq (or biomarkers discovered with it) can be used to monitor disease state and select the appropriate time to treat, given the benefits and costs of intervention. (**D**) Analysis of samples before and after treatment may reveal subsets refractory to a given therapy, as well as their biomarkers and mechanisms of resistance. (**E**) Because of its sensitivity, scRNA-seq might also be used in a clinical setting for detection of rare disease-associated cells (such as minimal residual disease), which would have been missed by bulk analyses.

## THE NEED TO PROFILE SINGLE DISEASE-ASSOCIATED CELLS

Ideally, personalized medicine would be guided by complete information about all disease-associated factors that might affect a patient’s treatment, from dysregulated genes and cells to lifestyle, diet, and environment. Initial steps toward the former have already begun: For example, genome-wide analyses of DNA have been used to pinpoint mutations that can be targeted by specific treatments in malignant disorders (*1*, *3*). However, cancer and other complex diseases commonly involve large numbers of genetic or epigenetic changes across several cell types, each of variable virulence and therapeutic value.

Identifying how these alterations collectively drive cellular dysfunction often requires a change of focus from individual DNA alterations to cellular pathways. Here, analysis of mRNA expression can complement DNA findings by highlighting aberrantly active gene modules. In addition, some mutations and copy number variations can be inferred directly from mRNA. There are already several diagnostic mRNA kits available for malignancies (*1*). However, because these analyze cells in bulk, their outputs mask differences between individual disease-associated cells. This can have disastrous consequences in cancer, where individual cells can drive drug resistance and metastasis. scRNA-seq could play a major role in overcoming this hurdle by providing a previously inaccessible degree of resolution to the characterization of cellular clinical isolates (Fig. 1, C to E).

## INTRODUCING SCRNA-SEQ FOR TRANSLATION

An intuitive way to think of scRNA-seq is to liken it to demographics. Whereas average population statistics (representing bulk genome-wide RNA-seq mRNA expression values) may tell us that an American (average cell) is around 37.8 years old, has about 1.14 children, and makes about $30,000/year, scRNA-seq comprehensively collects these same data points for each individual. By applying computational methods, we can identify distinct structures in our individual-resolved demographics (gene modules coexpressed across cells) that may reveal valuable groupings, for example, professions (cell types). Further, we can uncover additional attributes associated with these sets, such as professional training (pathways and genes), make predictions about their common connections (upstream drivers), and find unique identifiers for them (biomarkers). Thus, for a tumor, rather than seeing the average mRNA expression of some unknown mixture of cells, we can resolve each individual malignant, stromal, parenchymal, and immune cell, as well as the genes and pathways it expresses, with scRNA-seq. With this information, we can then find biomarkers and potentially devise interventions that specifically target each malignant subset (Fig. 1, B to E) (*4*–*6*).

Most scRNA-seq methods rely on a combination of reverse transcription and amplification, given the minute quantity of mRNA in a single cell (*7*). Amplification can be achieved by polymerase chain reaction (as in SMARTSeq2, Drop-Seq, Seq-Well, and 10x Genomics) or in vitro transcription (as in inDrop and CEL-Seq2). Several bespoke and commercial technologies have recently emerged, providing increased scale and decreased cost (*7*). For example, we recently described Seq-Well, a simple, portable, low-cost scRNA-seq platform aimed at translational research (*8*). Spatially resolved variants have also begun to appear and should help facilitate examination of the links between cellular phenotype and tissue location. We envision that spatial approaches, which are currently more experimentally taxing but also more akin to common clinical staining protocols and potentially applicable to archival tissues, will soon complement and enhance scRNA-seq findings from dissociated samples.

The initial applications of scRNA-seq to characterize diseased clinical materials have mainly focused on cancer. For example, one study used scRNA-seq to demonstrate considerable heterogeneity among malignant cells in a primary kidney cancer and its lung metastasis (*9*). Analyses of single-cell subsets identified a combination therapy that targeted two mutually exclusive pathways and was more effective than monotherapy in a patient-derived xenograft model. In oligodendroglioma, another scRNA-seq study supported a developmental tumor cell hierarchy wherein undifferentiated, stem cell–like cells could drive tumor growth and give rise to differentiated progeny (*6*). The authors speculated that targeting genes specific to the stem cell–like cells may have therapeutic potential. An additional important clinical application of scRNA-seq could be identifying rare cells, such as malignant cells that remain after treatment (minimal residual disease) (Fig. 1E). In parallel, other recent studies have shown the functional and clinical importance of multiple different nonmalignant tumor-associated cells (*4*, *5*), including stromal, parenchymal, and immune cells, as well as subsets thereof. These cells similarly vary greatly between patients, with diagnostic, therapeutic, and prognostic implications.

## FROM THE BENCH TO THE BEDSIDE

Accurately translating findings from scRNA-seq studies to clinical implementations of personalized medicine is complicated by measurement noise—both technical and biological—which affects how much information can be obtained using the method. One of the most important challenges is standardizing the handling of sensitive samples in clinical settings. Although protocols for processing biopsy materials have been developed (*4*–*6*, *9*), it is important to consider dissociation-induced artifacts and the degree to which any particular sample is representative of the overall diseased tissue. Spatially resolved methods, run in parallel or subsequent to dissociated scRNA-seq, may help address these concerns. Additional technical confounders include inefficiencies in mRNA capture and reverse transcription, as well as biases in amplification; biological ones include random and structured variability in the generation and degradation of mRNAs, and discrepancies between mRNA and protein expression. Some of these limitations can be addressed by experimental and computational means (*5*, *7*, *8*), such as the use of unique molecular identifiers to counteract amplification-induced artifacts. Still, before full clinical translation, studies will be needed to test the diagnostic, prognostic, and therapeutic predictions from scRNA-seq in large patient cohorts.

After such proofs of principle, several practical problems must still be solved to translate scRNA-seq to the clinic. Major points here include properly implementing a scRNA-seq workflow (optimizing sampling to preserve cellular states and ensure representativeness, establishing standardized library generation and sequencing procedures, training hospital staff, collecting proper consent, etc.) and handling the data (defining quality control metrics, removing technical artifacts, appropriately analyzing the data, etc.). Another challenge is ensuring interoperability between the data collected on different platforms because the rapid development of new scRNA-seq technologies will likely render existing approaches obsolete.

Together, these challenges imply that instituting experimental and computational pipelines at every clinic may be impractical. A potential experimental strategy to rapidly leverage the translational potential of scRNA-seq might be to have expert laboratories or centers rigorously perform assays on subsets of patients to discover diagnostic markers for different diseases that could then be more easily measured at most clinics using established assays. Because of the involvement of multiple cells and pathways— and, for single-cell readouts, the limited value of any single measurement given technical and biological noise—it is likely that combinations of these markers will be necessary. This may have an important advantage: Relative changes in the amounts of many markers can be more informative than absolute changes in individual ones (*4*, *6*–*10*); moreover, for single-cell measurements such as immunohistochemistry, changing patterns of marker coexpression may reveal more nuanced yet vital treatment-induced shifts (*4*, *5*). In some instances, scRNA-seq findings may also enhance the utility of population-level clinical assays by revealing the fundamental cell states and pathways needed to accurately deconvolve which cells comprise those population-level samples (*4*).

Another important hurdle is the preprocessing, analysis, and storage of the data. Therapeutic decisions will require user-friendly software, which allows understanding and visualization in physician-accessible formats. Although this may appear to be far from current clinical decision support systems, the bioinformatics principles for such software have already been developed and validated by functional and clinical studies (*2*). A feasible option may be to have centralized analytics (coupled with or distinct from select sample processing sites), which would enable each sample’s analysis to incorporate existing data and be reinterpreted as new data sets and clinical information become available. One illustrative model integrated genomic and clinical data from leukemia patients and showed evidence of both clinical relevance and reduction of costs (*10*).

There are also financial considerations. Although scRNA-seq may appear expensive, the severity and costs of some malignant diseases are strong motivators for considering powerful methods that can improve therapeutic efficacy. For example, treatment with new cancer drugs may exceed $10,000/month per patient. This sum does not include additional expenses associated with health care and productivity loss. In comparison, the prices for genomic analyses are rapidly decreasing. Although sequencing one human genome cost around $100 million in 2001, it is less than $1000 today. Recent experimental advances have similarly dropped the price of scRNAseq over 100-fold during the past decade, to cents per cell. Given these economics, getting buy-in from health care insurers will be an important but potentially feasible step.

A final challenge, as well as opportunity, is that an increasing number of patients may want information that will allow them to participate in therapeutic decisions (“participatory medicine”). This is likely to contribute to successful treatment outcomes but will require solutions to present and discuss increasingly complex medical data.

## CONCLUSION

Given the clinical needs and the economic challenges facing health care and drug development, we believe that there are strong incentives to clinically implement scRNA-seq for personalized medicine within the next decade. These efforts will likely focus first on cancer and then on other serious and prevalent maladies that require expensive treatments. Experiences from those and related applications and decreasing costs may pave the way to implementations for many common diseases. Although this will involve major challenges, it also presents excellent opportunities for treating and diagnosing cancer and other complex multifactorial diseases that cannot be missed.
